# Cement leakage causes potential thermal injury in vertebroplasty

**DOI:** 10.1186/1471-2474-12-116

**Published:** 2011-05-26

**Authors:** Po-Liang Lai, Ching-Lung Tai, Lih-Huei Chen, Nai-Yuan Nien

**Affiliations:** 1 Department of Orthopaedic Surgery, Chang Gung Memorial Hospital, Chang Gung University School of Medicine, Taoyuan, Taiwan; 2 Department of Chemical Engineering, National Tsing Hua University, Hsinchu, Taiwan; 3 Graduate Institute of Medical Mechatronics, Department of Mechanical Engineering, Chang Gung University, Taoyuan, Taiwan

## Abstract

**Background:**

Percutaneous vertebroplasty by injecting PMMA bone cement into the fractured vertebrae has been widely accepted in treatment of spinal compression fracture. However, the exothermic polymerization of bone cement may cause osseous or neural tissue injury. This study is thus designed to evaluate the potential risk of thermal damage in percutaneous vertebroplasty.

**Method:**

Twelve porcine vertebrae were immersed in 37°C saline for the experiment. In the first stage of the study, vertebroplasty without cement leakage (control group, n = 6) was simulated. The anterior cortex, foramen, posterior cortex and the center of the vertebral body were selected for temperature measurement. Parameters including peak temperature and duration above 45°C were recorded. In the second stage, a model (n = 6) simulating bone cement leaking into the spinal canal was designed. The methods for temperature measurement were identical to those used in the first stage.

**Results:**

In Stage 1 of the study (vertebroplasty of the porcine vertebral body in the absence of cement leakage), the average maximal temperature at the anterior cortex was 42.4 ± 2.2°C; at the neural foramen 39.5 ± 2.1°C; at the posterior cortex 40.0 ± 2.5°C and at the vertebral center, 68.1 ± 3.4°C. The average time interval above 45°C was 0 seconds at the anterior cortex; at the neural foramen, 0 seconds; at the posterior cortex, 0 seconds and at the vertebral center, 223 seconds. Thus, except at the core of the bone cement, temperatures around the vertebral body did not exceed 45°C. In Stage 2 of the study (cement leakage model), the average maximal temperature at the anterior cortex was 42.7 ± 2.4°C; at the neural foramen, 41.1 ± 0.4°C; at the posterior cortex, 59.1 ± 7.6°C and at the vertebral center, 77.3 ± 5.7°C. The average time interval above 45°C at the anterior cortex was 0 seconds; at the neural foramen, 0 seconds; at the posterior cortex, 329.3 seconds and at the vertebral center, 393.2 seconds. Based on these results, temperatures exceeded 45°C at the posterior cortex and at the vertebral center.

**Conclusions:**

The results indicated that, for bone cement confined within the vertebra, curing temperatures do not directly cause thermal injury to the nearby soft tissue. If bone cement leaks into the spinal canal, the exothermic reaction at the posterior cortex might result in thermal injury to the neural tissue.

## Background

Osteoporotic vertebral fractures are a major health care problem; they cause severe, debilitating back pain and consequent reduced physical function and have an enormous impact on quality of life [1]. Conservative management, which may include analgesics, bed rest, use of a brace, and rehabilitation, is indicated in patients who do not have neurological impairment. Surgery is indicated in patients who have symptoms that are refractory to conservative treatment modalities and in patients who have spinal instability and progressive neurological deficit. Open surgery is risky in older patients because of the poor quality of osteoporotic bone and co-morbidities.

Vertebroplasty, the percutaneous injection of polymethylmethacrylate (PMMA) into the fractured vertebral body, has been widely accepted as a treatment for painful osteoporotic vertebral fractures. Several studies suggest that immediate and sustained pain relief and restoration of vertebral body height are achieved through this procedure [2-4]. Vertebroplasty achieves its analgesic effect by preventing motion of the fractured fragments and by stabilizing the vertebral bodies.

PMMA is biocompatible and nonabsorbable. Because PMMA cement cures rapidly, reconstructions are mechanically stable within a few hours of surgery. However, the use of PMMA in vertebral augmentation is also associated with complications and risks. Possible cytotoxic and hypotensive effects of the PMMA monomer have been studied [5]. A possible cause of neurological deficit after intraspinal bone cement leakage is mechanical compression of the spinal cord [6-8]. Some authors have hypothesized that thermal injury could occur as a result of cement leakage occurred during vertebroplasty. It has been shown that the heat that develops during PMMA curing can damage not only the surrounding body tissue but also adjacent soft tissue, including the spinal cord, nerve roots, major vessels, and even lung parenchyma in the thoracic spine [9]. Wilkes reported cases of paraplegia that they attributed to thermal injury after PMMA injection into a vertebral body [10]. Uchiyama reported that protein damage and cell death in an animal model are aggravated when the temperature goes above 45°C [11]. However, literature that definitively addresses the effects of thermal damage during vertebroplasty is lacking. The present study was thus designed to illustrate the potential risk of thermal damage in percutaneous vertebroplasty in an in vitro animal model.

## Methods

In this study, porcine vertebral bodies were used as substitutes for human cadaveric spines. Ethics was approved by the review board of Chang Gung Memorial Hospital. We carried out the experiments in two stages.

### Stage 1. Vertebroplasty in porcine vertebral body

**Figure 1:**
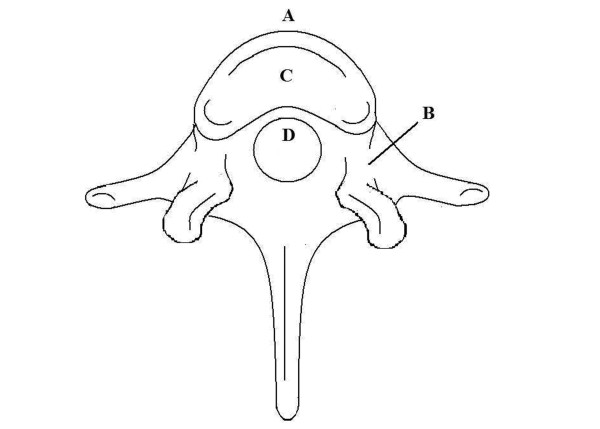
**Schematic drawings showing the locations of the four thermocouples**. (A) anterior cortex; (B) neural foramen; (C) center of vertebral body; (D) posterior cortex.

Six fresh vertebral bodies (T10-L1) harvested from fresh porcine spines were used. The vertebrae were disarticulated and their discs were excised. In order to standardize the baseline temperature, all specimens were immersed in 37°C saline in a water tank (Lipsh, SHANDO Co., USA) 30 minutes before injection of bone cement. Needle thermocouples (SE319, TECPEL Co., Taiwan) were used to record the temperature. The thermocouples were connected to a digital 4-input temperature data logger (Model: DTM-319, TECPEL Co., Taiwan) controlled by a personal computer. The temperature resolution was 0.1°C. To facilitate the attachment of thermocouples at the designated locations, the cortex of each vertebral body was penetrated using a 20-gauge needle. Thermocouples were inserted to a depth of 1.0 mm. After injection of PMMA cement, temperature changes at four locations in the vertebral body were measured simultaneously. The anterior thermocouple (1) was attached to the anterior cortex, halfway between the anterior margin of the superior endplate and the inferior endplate, representing the potential thermal effect on the great vessels. The foramen thermocouple (2) was attached to the inferior cortex of the pedicle at the point where the existing nerve root passes; this site was selected in order to determine the risk of thermal damage to the nerve roots. The posterior thermocouple (3) was attached to the posterior cortex, halfway between the posterior margin of the superior endplate and that of the inferior endplate; this site was used to estimate the risk of thermal damage to the spinal cord. The central thermocouple (4) was placed in the center of the cavity in which the PMMA cement was embedded and measured the core temperature of the PMMA cement. The placement of the four thermocouples is shown in Figure 1. Parameters including peak temperature and duration above 45°C were recorded.

A cavity was created in each porcine vertebral body by drilling to simulate a bone defect in the vertebral body. The entry point was the center of the superior endplate. The diameter of the drill was 12.7 mm, and the depth of penetration through the superior endplate was 30 mm. By calculation, the cavity volume was 3.8 mL. Each vertebra received 3.8 mL of PMMA cement (Simplex^® ^P, Stryker Inc., Ireland) embedded in the man-made cavity. The cement was mixed according to the manufacture's guidelines. Temperature changes were measured during the curing of the bone cement. Data were collected at 1 Hz; each specimen was measured for at least 16 minutes.

### Stage 2. Bone cement leakage model

**Figure 2:**
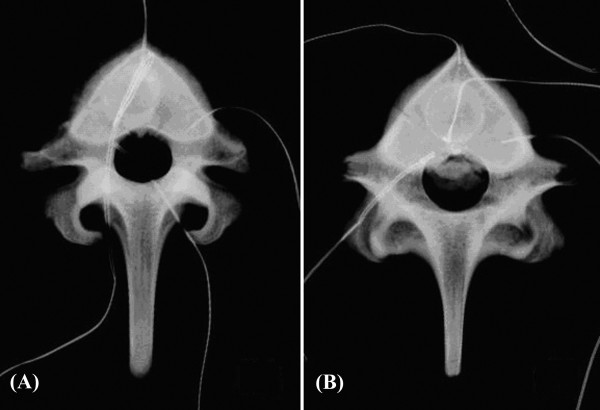
**X-ray photographs of porcine vertebra after vertebroplasty show (A) no leakage; (B) cement leakage into the spinal canal**.

A bone cement leakage model using the porcine spinal vertebrae was designed to simulate vertebroplasty with cement leakage. Another six fresh vertebral bodies (T10-L1) harvested from fresh porcine spines were used. The center point between the bilateral pedicles was designated as the leakage point of the posterior cortex. A 2-mm drill tip was used to penetrate the cortex to create the leakage point. Simplex^® ^P bone cement was injected into the cavity as in Stage 1. The posterior aspect of the vertebral body was kept in a horizontal position. The bone cement was injected continuously until the leaked bone cement flooded the margin of the vertebral body; the amount of injected bone cement depended on the amount of leaked bone cement. To measure the volume of the leaked bone cement, leaked cement was removed from the spinal canal via osteotomy. The volume of the leaked cement was measured by immersion in a 5-mL syringe containing 3 mL of water, and the elevated water level was measured. Using this method, we standardized the amount of leaked bone cement for each specimen (Figure 2). Needle thermocouples were connected to the four designated points described in Stage 1, and temperature measurement was carried out in a 37°C saline water tank. Parameters including peak temperature, duration above 45°C and time required to reach peak temperature, were recorded simultaneously during the entire process of PMMA cement polymerization.

### Statistical analysis

To evaluate the effect of cement leakage on temperature change at specific positions, a two-tailed Student's *t*-test was used to compare the highest temperature at each position between groups (stage 1 and stage 2). Differences were considered significant for p < 0.05.

## Results

All the specimens were porcine vertebrae, T10-L2; the average weight was 71.0 ± 5.1 g, and the mean length, width, and height were 26.1 ± 1.3 mm, 36.7 ± 1.6 mm and 37.8 ± 2.0 mm, respectively.

**Figure 3:**
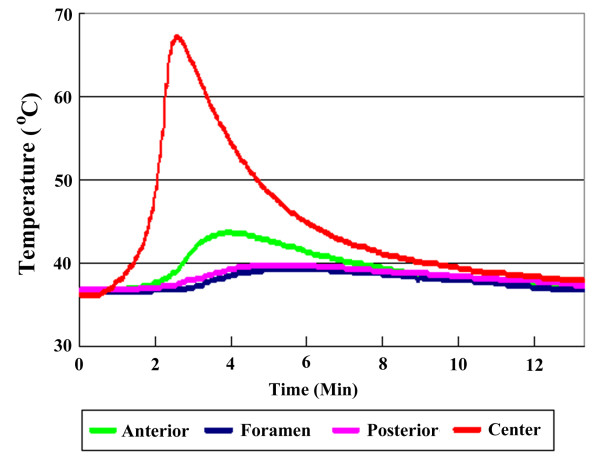
**Schematic image illustrating the duration of temperature changes at four different locations after vertebroplasty without cement leakage**. The temperatures at anterior cortex, neural foramen and posterior cortex remain below 45°C during the process of polymerization.

In Stage 1 of the study (vertebroplasty in the porcine vertebral body), the average maximal temperatures were 42.4 ± 2.2°C at the anterior cortex, 39.5 ± 2.1°C at the neural foramen, 40.0 ± 2.5°C at the posterior cortex and 68.1 ± 3.4°C at the vertebral center (Table 1). The average duration of temperature elevation above 45°C was 0 seconds at the anterior cortex, 0 seconds at the neural foramen, 0 seconds at the posterior cortex and 223 seconds at the vertebral center (Figure 3). Based on the results, temperatures around the vertebral body did not exceed 45°C except at the core of the bone cement.

**Table 1:** Highest observed temperatures at four locations within each specimen in Stage 1 (vertebroplasty without cement leakage) and Stage 2 (vertebroplasty with cement leakage) experiments.

	**Locations of thermocouples**							
	
	**Anterior**		**Foramen**		**Posterior**		**Center**	
				
**Specimen**	**Stage 1**	**Stage 2**	**Stage 1**	**Stage 2**	**Stage 1**	**Stage 2**	**Stage 1**	**Stage 2**
				
1	38.4	38.3	37.1	41.2	36.9	65.8	75.0	82.4
2	44.6	44.3	43.4	41.1	39.6	68.4	65.8	76.1
3	41.8	42.1	38.7	41.0	40.0	61.8	66.6	72.9
4	43.8	42.8	38.9	40.4	38.8	48.5	66.5	85.5
5	42.0	44.3	39.7	41.2	44.6	53.9	67.7	70.5
6	43.7	44.4	39.4	41.8	39.8	56.3	67.2	76.6
				
Avg.	42.4	42.7	39.5	41.1	40.0	59.1	68.1	77.3
SD	2.2	2.4	2.1	0.4	2.5	7.6	3.4	5.7
				
P-value	0.81		0.10		0.0001		0.007	

(°C)

**Figure 4:**
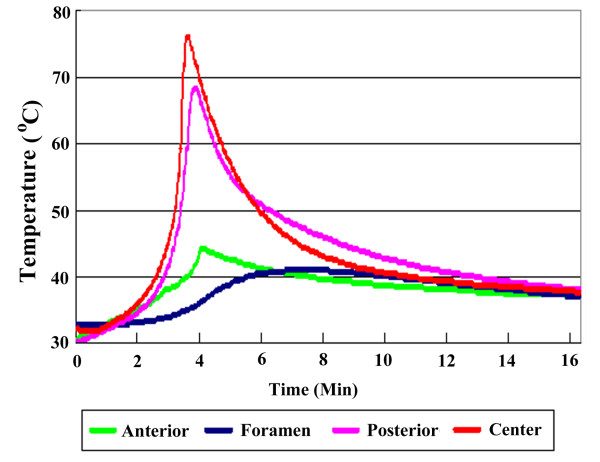
**Schematic image illustrating the duration of temperature changes at four different locations after vertebroplasty with cement leakage into the spinal canal**. The temperatures at the center of the vertebra and at the posterior cortex exceed 45°C.

In Stage 2 of the study (cement leakage model), the amount of leaked bone cement within the spinal canal was 1.18 ± 0.23 mL. The average maximal temperature was 42.7 ± 2.4°C at the anterior cortex; 41.1 ± 0.4°C at the neural foramen, 59.1 ± 7.6°C at the posterior cortex and 77.3 ± 5.7°C at the vertebral center (Table 1). The average duration of temperature elevation above 45°C was 0 seconds at the anterior cortex, 0 seconds at the neural foramen, 329.3 seconds at the posterior cortex and 393.2 seconds at the vertebral center (Figure 4). Based on the results, the temperatures exceeded 45°C at the posterior cortex and at the vertebral center.

Temperatures at the anterior cortex and the neural foramen did not exceed 45°C in vertebroplasty with or without cement leakage. However, at the posterior cortex, the peak temperature and duration above 45°C increased in the cement-leakage model. Thus, vertebroplasty with cement leakage resulted in conditions that are potentially thermally damaging to the surrounding soft tissue.

## Discussion

In clinical situations, it is not possible to standardize the amount of bone cement injected into the vertebral body, the distribution of bone cement within the vertebral body, and the distance between the cortex and the bone cement. The energy release by a bolus of cement is proportional to the volume of cement injected [12,13]. The distance between cortex and bone cement influences heat dispersion. In the present study, the amount of bone cement, the shape of the cavity and the distance between bone cement and cortex were standardized.

Experiments to study vertebroplasty, including quantitative injection of cement, placing thermocouples at designated locations, and deliberately leaking bone cement into the spinal canal, cannot be conducted in humans. However, numerous investigations using animal vertebral bodies as a substitute for cadaveric specimens have shown that animal vertebrae provide a good model for examining the mechanical characteristics and temperature profiles of vertebroplasty in human patients [14-16]. Zhao et al. [14] assessed the effects of surface treatment with a novel injectable strontium-containing bioactive bone cement (SrHAC) for vertebroplasty using porcine vertebrae. Their results revealed that the stiffness of the fractured spine recovered to 82.5% (p < 0.01) of the intact condition after cementation with surface-treated SrHAC. Aeble et al. [15] examined temperature profiles during vertebroplasty in a sheep model. These authors concluded that the spinal cord do not seem to be in danger of thermal damage during vertebroplasty if no leakage. In a recent study, Turner et al. [16] compared the compressive strength of adjacent motion segments and the histological response of vertebral bodies injected with calcium phosphate (CaP) or polymethylmethacrylate (PMMA) in a canine vertebroplasty model. Their results indicated no significant difference in vertebral body height and compressive strength between PMMA and CaP. However, unlike PMMA, CaP underwent resorption and remodeling, with vascular invasion and bone ingrowth.

Thermal damage from the exothermic curing of PMMA has been implicated as a factor in the development of bone necrosis around hip and knee implants. In vertebroplasty, the heat that develops during PMMA cement curing may damage not only the surrounding body tissue but also adjacent soft tissue including the spinal cord, nerve roots, major vessels, and even lung parenchyma in the thoracic spine. Toksvig-Larsen et al. used human cadavers to investigate temperature changes during PMMA cementation of a corpectomy defect and reported temperature increases of 4°C to 12°C on the external surfaces of the dura [17]. Thermal injury to neural structures is related to the increase in temperature and to the duration of exposure. Hyperthermia led to a dose-dependent decrease in motor and sensory function in a rat sciatic nerve treated in vivo with hyperthermia (43-45°C) for different times [18]. In an experimental study of spinal cord heating, Uchiyama concluded that, at 45°C or above, reduction of amplitude combined with shortened latency of nerve signals occurred [11].

Berman et al. suggested that PMMA bone cement be designed to limit intraoperative temperature maximums to less than 70°C, because, in a rabbit model, bone necrosis was consistently seen in histological sections of tissues exposed to temperatures greater than or equal to 70°C [19]. A sensitive vital microscopic method showed consistent and widespread bone tissue injury after heating to 50°C for one minute [20].

In a cadaveric study of vertebroplasty without cement leakage, the temperature at the spinal canal did not exceed 41°C and there was no evidence of injury to neural structures [21]. The posterior cortex of the vertebra acts as an insulator between heated bone cement and neural structures. Toksvig-Larsen et al. [17] reported that only a moderate temperature elevation on the surface of the dural sac provided that the posterior cortex of the vertebra was retained together with 0.5 cm of the spongious bone. The dorsal wall acts as a temperature barrier and prevents a significant increase in the temperate of the epidural space during cement curing. Aeblia et al. [15] concluded that the spinal cord does not seem to be in danger of thermal injury during vertebroplasty as long as there is no epidural leakage.

In the present study, the amount of bone cement, the shape of cavity and the distance between bone cement and cortex were standardized. In the stage 1 experiment, in which bone cement was confined within the vertebra, temperatures at the anterior cortex, neural foramen, and posterior cortex did not rise above 45°C. In the stage 2 experiment, in which bone cement leaked into the spinal canal, the peak average temperature of these areas was 59°C and the average temperature remained higher than 45°C for more than 5 minutes. The increase in temperature observed in this experiment shows that bone cement leaking into the spinal canal can potentially cause thermal injury to neural tissue. In addition to the commonly accepted concern that neural compression may occur due to space-occupying bone cement present in the spinal canal, the exothermic reaction of bone cement might result in neural tissue damage if there is direct contract between bone cement and neural tissue.

Leakage of bone cement during vertebroplasty will theoretically contribute to elevated temperatures at the anterior, foramen, posterior and center positions. Our data shows that the temperature differences observed in vertebroplasty with and without leakage are 0.3, 1.6, 19.1 and 9.2°C for the anterior, foramen, posterior and center positions respectively. The observed temperature difference is negligible at the anterior and foramen positions, significant at the posterior position and slightly different at the center position. Because the anterior and foramen positions were directly exposed to 37°C saline, the majority of the heat generated was transferred by convection through this medium. This resulted in the lack of significant temperature differences at the anterior and foramen positions. The posterior position was exposed to 37°C saline but concealed in the spinal canal; thus, heat dispersion at the posterior position was less efficient than at the anterior and foramen positions. Furthermore, the leaked bone cement directly contacted the thermocouple at the posterior position (and neural tissue in the clinical situation) causing significant temperature elevation. The center position was within the bulk of the bone cement and did not contact the saline solution; in this case, heat dispersion was by conduction through bone cement and bone instead of by convection through water. Heat accumulation caused a slight difference in temperature in vertebroplasties with and without leakage at the center position. Belkoff et al. [12] reported that internal temperature elevation in vertebroplasty caused osteonecrosis. Our study demonstrates that cement leakage into the spinal canal might cause neural thermal damage.

The obvious differences between clinical vertebroplasty and the in vitro porcine model point to several limitations of the present study. First, the specimens used are porcine vertebrae rather than human vertebrae. Second, vertebroplasty is simulated with man-made cavities instead of natural compression fractures. (However, the model in this study facilitated the amount and location of cement leakage.) Third, convective heat transfer due to blood perfusion was not accounted for in the current study but would be expected to reduce temperatures substantially *in vivo*. It is therefore possible that temperatures observed in this study overestimate the temperature changes that may occur *in vivo*. In turn, the heat convection effect of blood flow may not be as effective in the clinical situation because blood flow decreases with age [22]. Last, this study was conducted using disarticulated vertebrae instead of an en bloc spine. Temperature recordings from within the relevant soft tissues may have been more akin to in vivo temperatures.

## Conclusions

The results indicate that, for bone cement confined within vertebrae, curing temperatures of the cement typically used in vertebroplasty will not cause direct thermal injury to the nearby soft tissue. In case in which bone cement leaked into the spinal canal, the temperature at the posterior cortex was observed to rise to 59°C and to remain above 45°C for more than 5 minutes. Cement leakage into the spinal canal will potentially cause neural compression due to occupation of space by the cement as well as thermal injury due to the exothermicity of cement polymerization.

## Competing interests

This study was supported by a financial grant from Chang Gung Memorial Hospital (CMRPG381301). The funding source did not have any influence on the investigation.

## Authors' contributions

PLL set up the protocol, organized ethics approval, carried out the study and drafted the manuscript. CLT participated in the design of the study, the interpretation of the results and the drafting of the manuscript. LHC participated in the design of the study and helped with the analysis of data. NYN participated in carrying out the study and reviewing references.

All authors read and approved the final manuscript.

## Pre-publication history

The pre-publication history for this paper can be accessed here:

http://www.biomedcentral.com/1471-2474/12/116/prepub
